# Platelet-Rich Plasma in the Treatment of Subcutaneous Venous Access Device Scars: A Head-to-Head Patient Survey

**DOI:** 10.1155/2015/630601

**Published:** 2015-03-24

**Authors:** C. Eichler, M. Najafpour, A. Sauerwald, J. Puppe, M. Warm

**Affiliations:** ^1^Breast Cancer Center, Municipal Hospital Holweide, 51067 Cologne, Germany; ^2^Department of Gynecology and Obstetrics, Municipal Hospital Holweide, 51067 Cologne, Germany; ^3^Department of Gynecology and Obstetrics, Helios Klinikum, 53721 Siegburg, Germany; ^4^Department of Gynecology and Obstetrics, Hospital Dueren GmbH, 52351 Dueren, Germany; ^5^Department of Gynecology and Obstetrics, University of Cologne, 50931 Cologne, Germany

## Abstract

*Introduction*. Platelet-rich plasma (PRP) is a product widely used in sports medicine, tissue repair, and general surgery. A recent meta-analysis showed this product to be beneficial when introduced into a wound area, be it intra-articular (i.e., joint-injections) or direct introduction onto the wound surface. *Methods*. Between the years of 2012 and 2014 a questionnaire evaluating surgical outcome after port (venous access device) removal was answered by 100 patients in the control group and 20 patients in a PRP group, leading to a total of 120 patients in this single center, retrospective, subjective outcome evaluation. *Results*. No statistical difference was shown in postsurgical complication rates, postsurgical pain, decreased mobility, and overall quality of life. A significant difference was shown in overall patient satisfaction and the desire to further improve port area scarring. Results differed significantly in favor of the PRP group. Interestingly, approximately 40.2% of patients are dissatisfied with the surgical outcome after port removal in the control group. This result, though surprising, may be improved to 10% dissatisfaction when a PRP product is used. *Conclusion*. PRP products such as Arthrex ACP are safe to use and present an additional option in improving surgical outcome.

## 1. Introduction

Platelet-rich plasma (PRP) is a product widely used in sports medicine, tissue repair, and general surgery. A recent meta-analysis showed this product to be beneficial when introduced into a wound area, be it intra-articular [1] (i.e., joint) injection or direct introduction onto the wound surface [2–4]. Creaney and Hamilton have summarized the manner in which both platelets and plasma play an instrumental role in normal healing response [5]. In addition, literature indicates benefits in neurosurgery [6], as well as ophthalmic [7] and plastic surgery [8, 9]. Despite the fact that a variety of positive clinical data is available, initial enthusiastic reports may have to be reconsidered since some literature shows limited effectiveness [3]. It was the goal of this study to evaluate the clinical benefit derived from the use of PRP by using a patient questionnaire. 20 patients who had received PRP injections into the wound area after port system (venous access device) removal were compared directly to 100 retrospectively recruited patients who had not received PRP. It should be mentioned that since the removal of port systems is a significant cost factor of $1,485–3,325 [10], only 20 patients had received PRP due to the fact that the additional cost of this product is not covered in the German diagnosis related group (DRG) healthcare reimbursement system. Regardless of the study outcome, options for patients to receive this product are limited, due to the unique reimbursement situation. Although PRP has shown to be beneficial, the given baseline of extremely low clinical complication rates after port system removal would make it difficult to demonstrate benefits based only on factors such as postoperative pain, hematoma, seroma, and possible revision surgery. Also, literature only reports problems with in situ port systems or problems with different placement techniques [11, 12]. After port removal and completed chemotherapy, patients may once again focus on their physical appearance and self-image. In addition, patients often mention unsightly scarring, arm pain, and problems sleeping due to general discomfort in the surgical area. This remains true even after the venous access device has been removed. Since subjective patient evaluation of their port scar after removal showed a high degree of dissatisfaction (subjectively in everyday practice) we aimed to evaluate whether this could be addressed by offering patients the use of PRP. This of course, was conducted along with a variety of scar reducing measures which are common practice in the OB/GYN and oncoplastic surgical settings [13, 14].

## 2. Patients and Methods

Between the years of 2012 and 2014 all in-hospital port removals were asked to participate in a postsurgical survey. The survey was mailed to patients and included eight questions. Five of those questions were patient evaluated clinical factors, and three questions pertained to cosmetic results of the intervention. Clinical factors such as revision rates and seroma aspirations were collected form on-site patient files. This was a single center, retrospective analysis. In 2013 and 2014, twenty patients also received port system removal with the addition of PRP in the wound area. Patient age did not differ between the PRP and the control group, and all patients had received chemotherapy. None received radiation treatment on the ipsilateral side. The questions pertained to a timeframe within the first six months after port removal. A total of 150 patients were invited to participate in the control group. A written response was obtained from 100 patients. Thereafter recruitment was discontinued. This led to a total *N* of 120 patients (100 control, 20 PRP). Initial data analysis did not exceed more than 20 study group patients due to an additional cost factor associated with this type of intervention. Nonetheless, this procedure is currently being offered to patients and subsequent analyses will include more PRP patients.

*Questionnaire Endpoints.* Primary endpoints were arm pain, decreased mobility, difficulty in sleeping, quality-of-life decrease, and pain in the port area. All questions were evaluated on a five-point scale. An overview of questions and results may be found in Tables 1 through 3. Secondary endpoints were patient satisfaction with the results, the desire to improve result, and whether the results met patient expectations.

*Surgical Procedure.* The surgical procedure for port removal was the same in both groups. Absorbable suture material was used in all cases. All procedures were performed by experienced surgeons, in accordance with the gold standard for venous access device removal.

**Figure 1:**
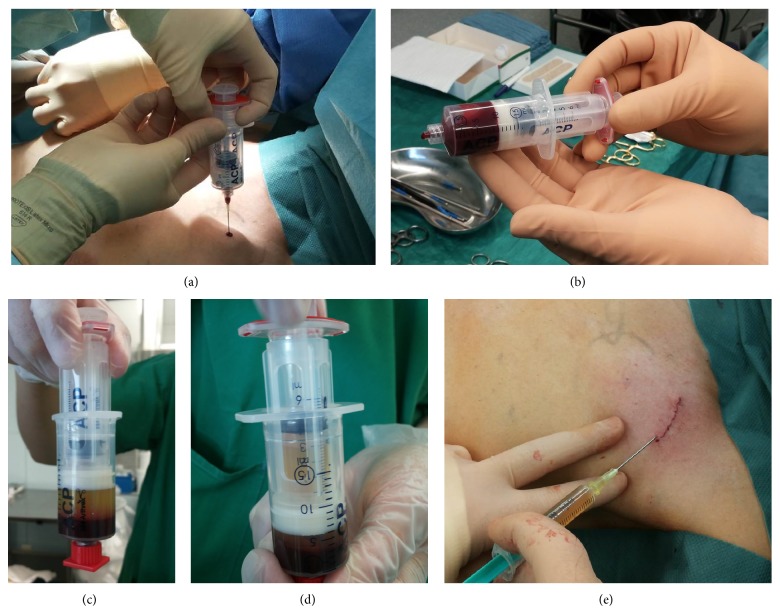
Shown are the preparation steps for the Arthrex ACP Double Syringe, PRP extraction system. (a) Showing the sterile extraction of autologous blood via the port system intraoperatively. (b) Showing 10 mL of whole blood in a double syringe system prior to centrifugation. (c) Showing the separation of PRP and erythrocyte layers after centrifugation. (d) Showing the harvesting of PRP via the double syringe system. (e) Showing the introduction of PRP into the wound area, after wound closure.

*PRP Preparation and Application*. PRP was prepared using the autologous conditioned plasma system (Arthrex ACP Double Syringe) by Arthrex [15]. Patient blood was extracted under sterile conditions during surgery via the port system. After centrifuge treatment, the double syringe system allowed sterile transfer of the PRP [16]. The PRP was then injected into the wound area before a sterile dressing was applied and after the intracutaneous wound closure was performed. See Figure 1.

*Informed Consent. *Written informed consent was obtained from all patients. A copy of the written consent is available for review. This study was conducted in accordance with institutional review board standard operating procedures. The application of a patient blood product was listed with the required municipal health agency. (i.e. Bezirksregierung Koeln, Dezernat 24: Oeffentliche Gesundheit, medizinische und pharmazeutische Angelegenheiten).

*Statistics. *Statistical analysis was performed using the VassarStats (Vassar College, Poughkeepsie, NY, USA) statistics program. Pearson's chi-squared tests and* t*-tests were used in order to evaluate significances when appropriate.

## 3. Results

There were no revision surgeries, hematoma, seroma, or wound infections in either group.

*Primary Endpoints.* Since eight categories were evaluated and patients were offered five options each, listing all results in this text body would be cumbersome. Thus the focus will be limited to the most frequently chosen options.

69.4% of the control group answered that they “never” suffered significant arm pain on the ipsilateral side. The same was true for 84.2% of the patients in the ACP group. This difference was not significant. Additionally, 22.4% of the control group and 15.8% in the ACP group reported “rarely” suffering from arm pain. A slight trend in favor of the ACP group is apparent, although significance could not be shown (*P* = 0.668). The same is true for the decreased mobility category where 68.4% and 84.2% reported never suffering from decreased mobility in the control and ACP group, respectively (*P* = 0.665). 70.1% and 90% of the patients did not report any sleeping difficulties in the control and ACP group, respectively (*P* = 0.559). A complete list of results is shown in Table 1.

56% (control) and 78.9% (ACP) reported “no” subjective decrease in quality of life (Qol) and only 20% (Control) and 5.3% (ACP) reported “very little” decrease in quality of life (*P* = 0.3316). While this is an unorthodox way to evaluate quality of life, we found that including a regular QoL questionnaire would drastically decrease the response rate due since more questions would have had to be answered by patients. Regarding pain in the port area within six months after removal 85.1% (control) and 95.0% (ACP) answered to experience with no pain at all. Again, a trend favoring the ACP group may be observed; however no statistical difference could be shown (*P* = 0.7871). An overview of all results may be found in Table 2.

*Secondary Endpoints. *While no statistical significance could be produced for the primary endpoints, although a trend favoring the ACP group could be shown, this does not hold true for the secondary endpoints. Results are listed in Table 3. When asked to evaluate general satisfaction with the scar area 27.8% and 12.4% of the control group claimed to be dissatisfied or rarely satisfied with the results. This contrasts with only 5% and 5% feeling accordingly in the ACP group. In turn only 54.7% claimed to be satisfied/very satisfied within the control group while 70% did so in the ACP group. These results differ significantly (*P* = 0.0167). When asked whether the patients would like to improve the surgical result 53.2% and 20.2% in the control group had the desire to improve the scar area. None of the patients in the ACP intended to do so (*P* < 0.001). Lastly, patients were asked whether the results met their expectations. This produced a fairly heterogeneous response for both groups. 56.4% (control) and 73.7% (ACP) reported results to meet or somewhat meet their expectations. However, 30.9% (control) versus 0% (ACP) were surprised by the extent of the scar tissue (*P* = 0.017).

## 4. Discussion

The use of PRP products has yielded good results in both orthopedic and general surgical areas [2, 17, 18]. Both in vivo and in vitro models show the benefit of delivering, for example, platelet released growth factors (i.e., PDGF-AB) into a wound area [19]. While PRP composition may vary significantly, interstudy comparisons are difficult. This is mirrored in the overall heterogeneity of available literature data. There is also no current literature available (PubMed, October, 2014) for the use of PRP in breast cancer patients in a port removal scenario.

Primary endpoints of this study showed that arm pain, decreased mobility, difficulty in sleeping, quality-of-life reduction, and pain in immediate port area did not differ significantly between the control and the ACP group. This may have several causes. The baseline in the control group sets a very high standard due to the fact that, using the example of pain in the port area, 93.6% (control) and 100% (ACP) of the patients reported to not experience any or very little pain in the port area after surgery. This is similar in all primary endpoint groups although a trend in favor of the ACP group may be observed. It may also be argued that given that this is a patient questionnaire-based analysis bias was produced as patients were informed that they would receive the PRP product which is known to improve the healing process. Regarding the primary endpoints it may therefore be stated that general port removal will result in a generally satisfied and pain-free patient even if PRP is not used. Nonetheless, offering additional options for wound care seems to further increase patient satisfaction. This in turn was shown by secondary endpoint analysis. Table 3 shows a fairly heterogeneous distribution within the satisfaction category of surgical outcome. For example, 27.8% of the control group patients seem to be completely dissatisfied with the scar tissue. While all above listed clinical parameters may be viewed as adequate, this level of patient dissatisfaction is not acceptable. In particular in a difficult postchemotherapy setting the additional psychological stress due to an unsatisfying body image may be problematic for overall patient morbidity [20, 21]. A somewhat surprising result was therefore that within the ACP group only two patients (10%) tend to be somewhat dissatisfied with their surgical result. One of them was a patient who had a very large port system scar prior to the intervention resulting in a dissatisfying evaluation of the postsurgical outcome. It should therefore be said that in order to circumvent overall dissatisfaction the additional use of PRP may be an option. These results are supported by 73% of the control group patients who reported that they would like to improve their surgical result.

This data has not yet been reported in other works but will have a direct impact on our physician-patient interaction prior to surgery. Before this data was available we felt that most patients did not mind a small port scar. Apparently this is not true for our study population (i.e., postchemotherapy breast cancer patients). Routinely offering a PRP product would increase the surgical costs by approximately $75 per intervention and is therefore only an option for private practitioners/clinics as the addition of this product is not part of the DRG reimbursement system. We may therefore conclude that clinical primary endpoints after port system removal display a more than adequate baseline regarding mobility, pain, and overall quality of life. Use of PRP does not significantly improve these tested areas. However, overall patient satisfaction is approved when PRP was used.

*Limitations. *While patient numbers in both the control and ACP group are adequate, an inherent bias in this study may tilt overall results in favor of the PRP group since patients were informed about PRP use prior to the intervention. A prospective, single-blind study would have to be performed in order to eliminate this factor.

## 5. Conclusion

Common clinical endpoints such as postsurgical complications (revision, infection, and seroma), postsurgical pain, or decreased mobility did not differ between both groups. Interestingly, 40.2% of patients are dissatisfied with the surgical outcome after port removal in the control group. This result, though surprising, may be improved to 10% dissatisfaction when a PRP product is used.

## Figures and Tables

**Table 1:** Results of the primary endpoints (arm pain, decreased mobility, and difficulty in sleeping) for the control group and the PRP (Arthrex ACP) group.

	Arm pain				Decreased mobility				Difficulty in sleeping			
	Control	%	ACP	%	Control	%	ACP	%	Control	%	ACP	%
Never	68	69.4	16	84.2	67	68.4	16	84.2	68	70.1	18	90
Rarely	22	22.4	3	15.8	24	24.5	3	15.8	16	16.5	1	5
Sometimes	4	4.1	—		2	2.0	—		10	10.3	1	5
Most of the time	3	3.1	—		4	4.1	—		3	3.1	—	
Always	1	1.0	—		1	1.0	—		—		—	

Total	98		19		98		19		97		20	
*P*	0.668				0.655				0.56			

**Table 2:** Results of the primary endpoints (quality-of-life decrease and pain in port area) for the control group and the PRP (Arthrex ACP) group.

	QoL decrease				Pain in port area			
	Control	%	ACP	%	Control	%	ACP	%
None	56	56	15	78.9	80	85.1	19	95
Very little	20	20	1	5.3	8	8.5	1	5
Some	5	5	1	5.3	2	2.1	—	
More than average	6	6	—		2	2.1	—	
A lot	13	13	2	10.5	2	2.1	—	

Total	100		19		94		20	
*P*	0.332				0.787			

**Table 3:** Results of the secondary endpoints (satisfaction with the result, desire for improvement, and expectations being met) for the control group and the PRP (Arthrex ACP) group.

	Satisfied with result				Would like to improve result				Result meets expectations			
	Control	%	ACP	%	Control	%	ACP	%	Control	%	ACP	%
Not at all	27	27.8	1	5	13	13.8	15	79	29	30.9	—	
Rarely	12	12.4	1	5	10	10.6	2	10.5	7	7.4	2	10.5
Sometimes	5	5.2	4	20	2	2.1	2	10.5	5	5.3	3	15.8
Most of the time	22	22.7	3	15	19	20.2	—		19	20.2	2	10.5
Always	31	32	11	55	50	53.2	—		34	36.2	12	63.2

Total	97		20		94		19		94			
*P*	0.02				<0.001				0.02			
